# Evaluation of an Innovative and Sustainable Pre-Commercial Compound as Replacement of Fish Meal in Diets for Rainbow Trout during Pre-Fattening Phase: Effects on Growth Performances, Haematological Parameters and Fillet Quality Traits

**DOI:** 10.3390/ani11123547

**Published:** 2021-12-14

**Authors:** Ümit Acar, Alessia Giannetto, Daniela Giannetto, Osman Sabri Kesbiç, Sevdan Yılmaz, Alessandro Romano, Rifat Tezel, Ali Türker, Kenan Güllü, Francesco Fazio

**Affiliations:** 1 Department of Forestry, Bayramiç Vocational School, Çanakkale Onsekiz Mart University, Çanakkale 17100, Turkey; 2 Department of Chemical, Biological, Pharmaceutical and Environmental Sciences, Messina University, 98166 Messina, Italy; 3 Department of Biology, Faculty of Science, Muğla Sıtkı Koçman University, Muğla 48000, Turkey; danielagiannetto@gmail.com; 4 Department of Animal Nutrition and Nutritional Diseases, Veterinary Faculty, Kastamonu University, Kastamonu 37100, Turkey; okesbic@kastamonu.edu.tr; 5 Department of Aquaculture, Marine Science and Technology Faculty, Çanakkale Onsekiz Mart University, Çanakkale 17100, Turkey; sevdanyilmaz@comu.edu.tr; 6 Ittinsect S.R.L., 00100 Rome, Italy; alessandro.ar.romano@gmail.com; 7 Department of Aquaculture, Faculty of Fisheries, Mugla Sıtkı Koçman University, Muğla 48000, Turkey; rifattezel@mu.edu.tr (R.T.); aliturker@mu.edu.tr (A.T.); kenangullu@mu.edu.tr (K.G.); 8 Department of Veterinary Sciences, Messina University, 98166 Messina, Italy; francesco.fazio@unime.it

**Keywords:** ITTINSECT™ APS V1, alternative protein source, fish meal replacement, rainbow trout, gene expression, growth performance

## Abstract

**Simple Summary:**

The study aims to define the potential and sustainable use of pre-commercial product ITTINSECT™ APS V1 as fish meal replacement in the diets of rainbow trout *Oncorhynchus mykiss*. For this, a 60 days feeding trail was performed by using diets with different replacing rate of fishmeal (0 (ITTM_0_), 25 (ITT_25_), 50 (ITT_50_), 75 (ITT_75_) and 100 (ITT_100_) %) with ITTINSECT™ APS V1. At the end of the feeding trial, significantly higher growth performance was observed in the group fed with the diet with a 25% and 50% substitution of fish meal by ITTINSECT™ APS V1. Moreover, the growth-related gene expressions analyzed in muscle tissue had significantly higher gene expression levels for these two diets (25% and 50% substitution) when compared to the control. The hematology values were found to be identical, whereas other parameters (such as serum total protein, globulins and glucose levels, or some immune-related gene expression) had different results among the experimental groups. In conclusion, replacement of fish meal with up to 50% ITTINSECT™ APS V1 in diets for rainbow trout is suggested in order to achieve the best growth performance in rainbow trout and enhance sustainable aquaculture practices.

**Abstract:**

The aim of the study was to determine the potential and sustainable use of pre-commercial product ITTINSECT™ APS V1 as a major protein source in rainbow trout (*Oncorhynchus mykiss*) diets. A 60-day feeding experiment was conducted to potentially use ITTINSECT as fish meal replacement in the diets of rainbow trout. Five isonitrogenous in dry matter (38% crude protein) and isolipidic (15% crude lipid) diets were produced: a control diet (fishmeal-based) (ITT_0_) and four experimental diets replacing fishmeal by 25 (ITT_25_), 50 (ITT_50_), 75 (ITT_75_) and 100 (ITT_100_) %, with ITTINSECT™ APS V1. Triplicate tanks, containing 15 fish each (65.81 ± 1.26 g), were hand-fed to apparent satiation twice every day during the experiment. At the end of the feeding trial, significantly higher growth performance was observed in the group fed ITTM_25_ and ITTM_50_ diets. This performance was supported by growth-related gene expressions analyzed in muscle; significantly higher GH and IGF-I genes expression levels were determined in ITT_25_ and ITT_50_ when compared to control (ITT_0_) (*p* < 0.05). While no significant differences were found between the hematology values (*p* > 0.05), serum total protein, globulins and glucose levels were significantly different between experimental groups (*p* < 0.05). In addition to this, the immune-related genes such as TNF-α, IL8 and IL1-β expression levels were determined to be significantly different (*p* < 0.05). In conclusion, in order to achieve the best growth performance in rainbow trout and enhance sustainable aquaculture practices, replacement of fish meal with up to 50% ITTINSECT™ APS V1 in diets for rainbow trout is suggested.

## 1. Introduction

Fish meal (FM) is a costly raw material that increases the feed costs in aquaculture [1,2]. As a result, research is being conducted to determine the feasibility of using alternative plant or animal sources in fish feeds, which will allow for more cost-effective feed production [3,4]. The rapid increase of the aquaculture industry worldwide also increases the need for such feed. However, it is very difficult to meet the current demand by producing a fish-meal-based feed made of wild-caught fish. On the other hand, the use of these species as human food adversely affects the future of the aquaculture feed industry [5]. In order to ensure sustainable fish production, the use of vegetable protein sources in feeds has become very common. However, in carnivorous species such as rainbow trout, even soy, the richest plant-based protein is problematic regarding flavor, antinutritional factors, and the ability to meet daily nutritional needs [6,7]. The use of plant sources in aquaculture feeds is still seen as an unsolved problem [8]. Apart from vegetable protein sources, the European Commission/Annex II of Regulation 2017/893 of 24 May 2017 approved the use of insect meal as an ingredient in fish feeds. Thus, studies using some insect species in fish feed are available in the literature [9,10,11,12]. Apart from obtaining fish living in nature by hunting, insect meal can be easily grown under controlled conditions and has low ecological impact and ammonia release. It is also very low in consumption as human food [13,14]. In addition, thanks to its balanced protein, amino acid and fatty acid content, insect meal is considered to be a suitable protein source that can be used instead of fish meal in aquaculture feeds [9].

In general, the observation of growth response is not enough to identify the impact of feeding on the health of fish. The changes in blood parameters that are based on feeding activities give a more reliable indication of the health state of fish [15,16].

The ITTINSECT™ APS V1 product line is a pre-commercial, sustainable aquaculture feed especially developed to have zero impact on the ocean. The Italian company Ittinsect s.r.l. is developing the product with the inclusion of alternative protein sources that improve the organic performance of finfish species. The product contains high percentages of yellow mealworm (*Tenebrio molitor*) and black soldier fly (*Hermetia illucens*) meals, processed with different methods to balance proteins, lipids and chitin. The nutritional profile of the feed was completed with the inclusion of plant- and animal-based nutrients derived from upcycled by-products, with the aim of replicating the nutritional profile of fishmeal.

As of today, there little scientific information regarding the effects of aquaculture feeds including a major insect protein component. Furthermore, to the best of our knowledge, it is the first time that a feed ingredient has been engineered using more than one species of insect at the same time and using a different processing method for each species of insect.

Thus, the present study aims to report the first results on the effects of the ITTINSECT™ APS V1 (ITTM) ingredient on growth performance, serum biochemical variables, haematological parameters, dietary chemical composition, proximate composition fillet amino acid and the fatty acid contents of rainbow trout (*Oncorhynchus mykiss*).

## 2. Materials and Methods

### 2.1. Experimental Diets, Sustainability and Economic Assessment

Among the insect ingredients, 50% of weight was black soldier fly and 50% of weight was meal worm. Black soldier fly is defatted, while mealworm is full-fat. Full-fat ingredient was used to add a relevant lipid percentage to the overall formula, as well as to reduce the economic and environmental cost of de-fatting insect meals. In a full-fat insect meal, the relative percentage of chitin is smaller than in a de-fatted insect meal, hence more inclusion of insect by weight could be used. Both black soldier fly and mealworm received an enzymatic pre-treatment to increase nutrient absorption. The enzymatic process cannot be given in detail because it is under consideration for a patent. The Ittinsect APS mix was complemented with lyophilised yeasts collected from brewery by-products.

The experimental diets were produced in the laboratory. All dry ingredients were carefully mixed with a laboratory feed mixer for diet preparation. The mixtures were primed with tap water to yield a suitable pulp. Wet diets were assembled into 3-mm pellets by laboratory scale pellet mill (Pellet Mill, SYSLJ-1, Henan, China), dried at 40 °C in a drying cabinet for 18 h and stored at −20 °C until use. Five isonitrogenous (38% protein) and isolipidic (15% crude lipid) diets in dry matter were produced for the experiment. The control diet was FM-based, and four experimental diets replaced FM with ITTM by 25%, 50%, 75% and 100%. The formulation, feed cost and nutrient composition of the experimental diets and major protein components are shown in Table 1. The prices were set according to the local market retail prices (at the current time) of diets. These prices (US $/kg) are as follows: herring FM, 2.035; soybean meal, 0.44; ITTM 1.61; wheat meal, 0.24; corn starch, 0.24; fish oil, 2.05; and vitamin-mineral premix, 0.50.

Accordingly [17], the following formulae were used to compute the relative utilization values of marine-derived feed components, fish meal (FMU) and fish oil (FOU):

FMU (g/kg of fish gain) = fish meal share in the diet (g/kg) × (feed intake (g)/body weight gain (g))
FOU (g/kg of fish gain) = fish oil share in the diet (g/kg) × (feed intake (g)/body weight gain (g))

The currency type for economic evaluations is the US dollar ($). The economic conversion ratio (ECR) was calculated using the following equation:

The economic conversion ratio (ECR) and economic profit index (EPI) were computed by [18] to measure the economic relative efficiency and advantages of the tested diet cost of feed per unit of fish gain.
ECR ($/kg of fish gain) = (feed intake (g)/body weight gain (g)) × cost of feed ($/kg)
EPI ($/fish^−1^) = [weight gain (kg) × selling price (4 $ kg^−1^)] − [weight gain (kg) × diet price ($ kg^−1^)]
Rainbow trout sale price calculated at 4 $ kg^−1^.

### 2.2. Fish and Feeding Trial

A 60-day experiment was carried out on 225 rainbow trout obtained from a private fish farm (Çanakkale, Turkey). At the begining of the experiment, fish were weighted individually (65.81 ± 1.26 g) and divided into fifteen fiberglass tanks of 0.25 m^3^ equipped with recirculating aquaculture system (RAS), with three replicates per experimental diets. The experimental fish were acclimated to the experimental tanks for one week and fed with extruded 3 mm commercial rainbow trout diet (Agromey, Turkey) prior to the start of the experiment. The experimental fish were fed two times a day until apparent satiation. During the acclimatization and experimentation periods, the fish were held at ambient temperatures of 12 ± 1 °C, dissolved oxygen of 7.02 ± 0.78 mg L^−1^ and pH of 7.22 ± 0.46.

### 2.3. Chemical Analyses of Feed and Fillets

Feed and fish (five fish per tank) samples were analyzed for proximate composition according to AOAC [19] at the end of trial. Following AOAC’s methods [18], dry matter (AOAC, 934.01), Ash (AOAC, 942.05) and proteins (N × 6.25; AOAC, 955.04) were determined. All experimental diets and fish fillet samples for amino acid (AA) analysis were first freze-dried and homogenized by mortar. Homogenized samples were hydrolyzed with 6 N HCl (0.1 g 20 mL^−1^) 24 h at 110 °C under nitrogen atmosphere [20]. Amino acids were determined by Shimadzu LC-MS 8040 (Shimadzu corporation Kyoto, Japan) [21]. The separation column Zorbax Eclipse AAA 4.6 × 150 mm, 3.5 µm was used in the analysis. Mobile phase A was composed of formic acid, water 1/1000 (*v*/*v*) and mobile phase B formic acid, methanol 1/1000 (*v*/*v*). For flow rates of 1 mL min^−1^, the column temperature was set at 40 °C, and the injected sample volume was 0.2 μL. The certified L-amino acid mix standard (Sigma-Aldrich 79248) for calibration was used for AA analysis [22].

### 2.4. Blood Sampling and Analyses

Blood was collected from the fish at the end of the 60-day trial from a total of 15 fish per group, 5 from each tank. The fish were randomly selected from the test tanks and instantly fainted in a bucket containing a natural stunner and commonly used clove oil (20 mg L^−1^) [23]. A 2.5 mL plastic syringe was used to sample blood from the caudal vein. Blood samples were divided into K3EDTA and cellular serum tubes for hematological and serum biochemical analyses. The gland-plated blood was centrifuged at 5000× *g* for 10 min for serum analysis. The serum was then stored at −80 °C before the analyses. Red blood cells, haematocrit and haemoglobin concentration were determined by using the method of [24]. RBC was counted with a Thoma hemocytometer with the usage of Dacie’s diluting fluid. The haematocrit was determined by using a capillary hematocrit tube. The haemoglobin concentration was determined with spectrophotometry (540 nm) by using the cyanomethahemoglobin method. Biochemical indices such as glucose (GLU), total protein (TPROT), albumin (ALB), triglyceride (TRI), cholesterol (CHOL), alkaline phosphatase (ALP), glutamic oxaloacetic transaminase (GOT), glutamic pyruvic transaminase (GPT) and lactate dehydrogenase (LDH) in serum were analyzed using bioanalytical test kits (PG Instruments, Leicestershire, UK).

### 2.5. Gene Expression Analysis

To determinate growth-related gene expressions such as *GH*, and *IGF-1* in muscle tissue and immune-related genes such as *TNF*-α, *IL-1ß* and *IL-8* in liver tissue, samples from three fishes of each tank were kept in RNAlater (Thermo Scientific, Waltham, USA) solution at −20 °C for total RNA extraction for growth- and immune-related gene expression studies. Real-time qPCR data was analyzed using the efficiency corrected relative expression method with β-actin as a reference gene. The GeneJet RNA purification kit was used to isolate the RNA (Thermo Scientific, Waltham, USA). A MultiskanTM FC Microplate Photometer (Thermo Scientific, Waltham, USA) was used to assess the quality of extracted RNAs. DNase-I (Thermo Scientific, Waltham, USA) was used to separate DNA from RNAs, and RevertAid H Minus Single Strand cDNA Synthesis Kit was used to create cDNA (Thermo Scientific, Waltham, USA). NCBI web site; mRNA sequences of the β-actin; GH, IGF-I, IL-8, TNF-α, and IL-1β genes, which are unique to rainbow trout; and FastPCR 6.0 package software were used to create primers [25]. Table 2 lists primer sequences, total base length and gene bank numbers. Real-time PCR (Bio RAD, ABD) device was used with a view to identify the differences in gene expression levels of the experimental groups. PCR analysis was conducted with PCR mix, Maxima SYBR Green qPCR Master Mix and ROX Solution (Thermo Scientific, Waltham, USA). Analysis of real-time PCR results was conducted via CFX Manager 3.1 software. Proportional changes in mRNA expression levels of target genes were calculated through 2^−ΔΔCt^ method based on cycle thresholds (Ct) of amplification curves obtained following amplification process comprising denaturation, annealing of primer and chain extension [26].

### 2.6. Calculations

Growth performance and feed utilization were calculated using following equations:

FCR (feed conversion ratio) = feed consumed/weight gain(1)

RGR (relative growth rate %) = ((final wet weight − initial wet weight)/initial wet weight) × 100(2)

SGR (specific growth rate %/day) = ((ln final wet weight − ln initial wet weight)/days) × 100(3)

### 2.7. Statistical Analyses

All values were presented as means ± SD and analyzed by SPSS 17.0 software (SPSS Inc., Chicago, IL, USA). One-way analysis of variance (ANOVA) followed by post-hoc Tukey’s test was used to reveal whether there were differences in the measured parameters among the treatments. All analyses were performed at a significance *p* level of 0.05.

## 3. Results

### 3.1. Growth Performance

All the test diets were comparable in terms of nutrients, such as protein, lipid and amino acid (AA) contents for rainbow trout. The AA composition of ITTINSECT and diets is presented in Table 1.

Growth parameters and survival rates are presented in Table 3. No mortality was observed during the experiment. After 60 days of feeding, growth performance, FCR and SGR values showed clear dietary supplementation effects of ITTINSECT (*p* < 0.05). The ITTM_25_ and ITTM_50_ groups showed the best values for WG, FCR and SGR, compared to the other experimental groups (*p* < 0.05). The relative FM usages in diets decreased per kg of fish gain. On the contrary, although FO usage in the diets did not change, it was found to be higher in groups due to low weight gain. Regarding the economic analyses, the different dietary levels of ITTM affected the diets cost and economic parameters such as ECR, EPI and PRO (*p* < 0.05) (Table 3).

### 3.2. Fillet Proximate Composition and Amino Acid Profile

The fillet chemical and AA compositions of the experimental fish fed with the experimental diets are shown in Table 4.

Based on the chemical composition crude protein, ash and moisture were not affected by the treatment. The inclusion of ITTM significantly increased crude lipid contents in all the four diets containing ITTM_25_, ITTM_50_, ITTM_75_ and ITTM_100_, compared to the ITTM_0_. Additionally, the high lipid content of the fillet in fish of the four ITTM diets did not show notable differences among them. Fillet essential amino acid (EAA) and non-essential amino acid (NEAA) content of rainbow trout fillets are presented in Table 4. Among EAAs of fillet leucine, lysine and valine showed significant decreases in the high dietary inclusion levels of ITTM (75 and 100%) (*p* < 0.05). For NEAAs such as glutamine, proline and serine showed similar trends and tended to decrease due to increase of ITTM levels in diets for rainbow trout (*p* < 0.05).

### 3.3. Hematological and Serum Biochemical Parameters

Hematological and serum biochemical parameters results are given in Table 5.

The RBC count, Hct and Hgb concentration in all experimental groups were not found significantly different (*p* > 0.05). Serum TRIG, CHOL, and ALB values showed no significant changes (*p* > 0.05), but TPROT, and GLO, a serum protein, tended to decline when replacement levels exceeded 75 percent, while significantly lower serum glucose level was determined in the group fed with ITTM_75_ diet compared control group (*p* < 0.05).

### 3.4. Gene Expression

**Figure 1:**
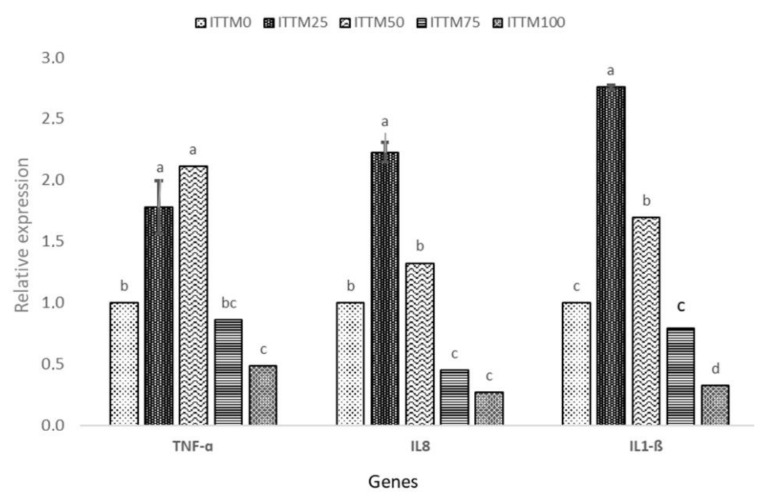
Relative gene expression (mean ± SD) of TNF-α, IL-8 and IL-1β in the liver of rainbow trout fed with experimental diets. Values with common superscripts (a,b,c) are not significantly different (*p* > 0.05).

**Figure 2:**
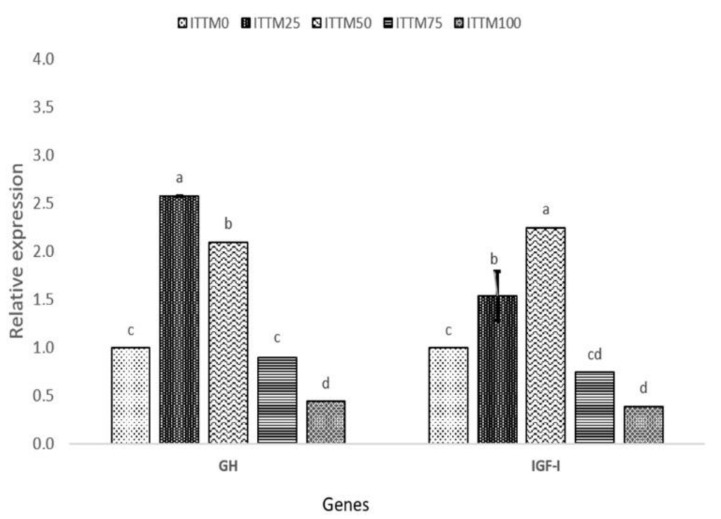
Relative gene expression (mean ± SD) of GH and IGF-I in the muscle of rainbow trout fed with experimental diets. Values with common superscripts (a,b,c) are not significantly different (*p* > 0.05).

Changes in gene expression responses of the fish after the 60-day feeding experiment with ITTM supplemented and control diet are presented in Figure 1 and Figure 2.

The fish fed with ITTM_25_ and ITTM_50_ diets showed a significantly higher expression of GH, IGF-I, TNF-α, IL-8 and IL-1β in the muscle and liver tissue compared to the other experimental groups (*p* < 0.05). Significant down-regulation was shown in fish fed with ITTM_75_ and ITTM_100_ diets (*p* < 0.05).

## 4. Discussion

It is well known that fish farming relies heavily on marine captures for the nutrition of carnivorous fish species. However, such fisheries are typically not sustainable, and an increased demand for livestock and aquaculture feeds has resulted in a rapid reduction in fishmeal availability. This has led to a rapid increase in prices, where feed expenses already absorb between 40 and 70 percent of the cost of farmed fish [9]. Therefore, in the past years several alternatives to FM have been explored, among which insect biomass stands out as a promising alternative protein source that could replace FM. Insects rich in amino acids, lipids, vitamins and minerals leave a significantly smaller ecological footprint, and some species even show anti-fungal activity and/or anti-fungal peptides that may increase the shelf/life of insect-containing feeds [9]. In the current study, a basic economic analysis of feed production revealed that raising the content of ITTM in fish diets reduced the diet cost from 1.36 to 1.17 US$ per kg diet (Table 1). Feed price, feeding rate, stocking density, fish size, fish production and fish sales are all important factors in determining the best profit in aquaculture [27]. According to the findings of this study, using ITTM as a FM replacer in fish diets up to 50% has a favorable economic impact, lowering feed costs by 6.62%. Similar results were reported by [28] when black soldier fly larvae meal was used as a FM replacer in European seabass (*Dicentrarchus labrax*) diets. The present experimental results showed the high environmental sustainability of ITTM use in rainbow trout diets. Due to improved feed utilization and growth performance in ITTM_50_ diet in the present study, FM use was decreased for rainbow trout production. Previous study on Siberian sturgeon using black fly meal reported reduction in FM up to 70% and FO up to 96.5% in fish diets [29].

The results of the growth performance showed that the inclusion of ITTM in diet for rainbow trout up to 50% did not lead to any adverse effect on growth performance. Previous studies reported that the use of insect-based meals such as yellow mealworm and black soldier fly as an alternative protein source for FM alone or in combination did not affect growth performance of fish species such as channel catfish (*Ictalurus punctatus*) and blue tilapia (*Oreochromis aureus*) [30]. Similarly, [31] demonstrated that defatted black soldier fly could entirely replace FM in Jian carp (*Cyprinus carpio* var. *Jian*) diets. According to [32], up to 19.5% of pre-pupae meal of black soldier fly can be successfully used in diets as a raw material of juvenile European seabass with no adverse effect on growth performance, feed utilization, apparent digestibility coefficients or digestive enzyme activity. Growth performance in rainbow trout was unaffected by dietary addition of 25% black soldier meal, while it was decreased by dietary inclusion of 50% black soldier fly meal [33]. Kroeckel et al. [34], on the other hand, found a substantial decline in turbot (*Psetta maxima*) growth performance for all FM substitutions with black soldiers fly meal. Replacement of FM with diets including yellow feedworm up to 100% did not result in any adverse effect on growth performance except for the case reported by Chemello et al. [35]. The present study is the first attempt to evaluate the ITTM effects as partial or total replacement of FM. The inconsistences in the results provided by Chemello et al. [35] could be due to the differences in fish species and sizes, insect species and cultivation area, and insect processing techniques [36].

In the present study, considering the proximate composition of the fillet of rainbow trout, no significant differences in moisture, protein and ash content emerged by inclusion of ITTM in to the diets. Nevertheless, a significant increase was obtained in fillet lipid composition with the increase of ITTM level in the diets. Melenchónet al. [37] reported similar results for rainbow trout fed with black soldier fly meal and stated that the substitution of fish meal used in rainbow trout diets with a low percentage of commercial black soldier fly meal tended to increases the amount of fillet lipid level. On the contrary, Belforti et al. [38] reported significant decreases of total lipids content, with increasing inclusion of *Tenebrio molitor* larvae meal diets for rainbow trout. The fluctuations in total lipid levels in the fish body can be connected to variations in synthesis alterations, muscle deposition rate and/or various growth rates in lipids [39]. In addition to this, Table 4 presents the effects of the experimental diets on the aminoacid composition of rainbow trout flesh. In the fillet of fish that consumed the ITTM_50_ diet, there was a substantial drop in amino acids such as Leu, Lys and Val, but they stayed constant in the ITTM_25_ feeding group compared to the ITTM_0_ group. A reducing trend in the same amino acids has been reported in rainbow trout fillet that were fed with mealworm larvae meal [40].

Hemato-biochemical variables are frequently utilized to assess nutritional status, health conditions and fish adaptability to the outside environment [41]. In this study, any changes in the values of several hematological parameters were observed after ITTM inclusion in diets for the rainbow trout (*p* > 0.05). Similarly, Abdel-Tawwb et al. [28] and Tippayadara et al. [42] reported that replacement of FM by black soldier fly meal had no effects on European sea bass and Nile tilapia (*Oreochromis niloticus*) hematological parameters, respectively. An increase in serum total protein, albumin and globulin levels is a good indicator of the health and immunological status of fish [43,44]. Insect meal chitin has been related to animal immunopotentiation, and this has been shown in fish and shrimps [11,45,46]. Although, there were significant changes in the total serum protein, and in globulin from ITTM_0_, ITTM_25_, ITTM_50_ and ITTM_100_ in the current investigation, a modest improvement in fish given low ITTM levels (25%) was seen, indicating a potential immunostimulation of ITTM owing to chitin content. The presence of chitin has been reported to play a role also in triglyceride hydrolysis [47]. Accordingly, in the present study no significant differences among all groups were observed in terms of serum cholesterol and triglyceride values. Although some studies reported significantly lower cholesterol levels in fish fed with insect meal than in those fed with fish-meal-based diets [33], our study found no obvious change in cholesterol of the ITTM groups compared to the control group. These disparities may be also due to the different fish sizes, insect species, insect matrix and processing methods [38]. Cholesterol metabolism in fish differs from that in terrestrial animal models, and there are several processes involved in the regulation of cholesterolemia that require more investigations [48]. Similar results for serum cholesterol levels were reported by Lu et al. [49] in grass carp (*Ctenopharyngodon idella*) fed with black soldier fly larvae meal.

In salmonids, GH plays an important function in somatic development by promoting a synthesis of protein, feed intake and feeding efficiency [50,51]. Furthermore, GH and IGF-1 participate in many other fish activities, including feeding, predator prevention and osmoregulation [52]. In this study, and in line with growth performance results, differences were observed in the expression of GH receptors or IGF-I transcripts among diets. In our study, rainbow trout received their nutritional requirements according to apparent satiation, but fish fed ITTM_75_ and ITTM_100_ diets showed lower feed utilization than other diets. The lower expression of GH and IGF-I in muscle of fish fed with these two diets possibly indicates a lower level of nutrition and growth rate. Immune-related genes, such as TNF-α, Il-8 and IL-1β, showed a significant up-regulation among the test diets ITTM_25_ and ITTM_50_. Similar findings were reported by Hender et al. [53] and Zarantoniello et al. [54] in Zebrafish (*Danio rerio*). Additionally, Kumar et al. [55] found an up-regulation in kidney of rainbow trout when fish were fed with up to 16% black soldier fly supplemented diets. Although, chitin in aquafeeds with positive effects on the fisheries immune system is found in all insect-based diets [10] and may cause intestinal inflammation and decline in the assimilation of nutrition (>30%). The increased incorporation of ITTM in rainbow trout liver in the present investigation has shown significant amounts of chitin that have detrimental impacts on the expression of immune-related genes.

In conclusion, this study demonstrated that at least 50% of FM protein could be replaced with ITTM without any adverse effects on growth performance, fillet composition, blood parameters, growth and immune-related gene expression of rainbow trout. Despite this, it is important to emphasize that the complete life-cycle of rainbow trout from juveniles to adults requires long-term research to produce an increase in ITTM levels to provide additional information about immune-relevant gene expression.

## Figures and Tables

**Table 1:** Ingredients (g kg^−1^), composition of the experimental diets and essential amino acids (AA) profile (as % of protein) in anchovy fish meal (AFM), ITTINSECT^®^ meal (ITTM), and experimental diets containing graded replacement levels of AFM with ITT on protein unit basis as well as amino acid requirements for Rainbow trout as (% of dietary protein).

Ingredients Composition (g kg^−1^)			ITT_0_	ITT_25_	ITT_50_	ITT_75_	ITT_100_
Fish meal ^1^			470	352.5	235	117.5	-
ITTINSECT^®^ meal			-	117.5	235	352.5	470
Soybean meal ^2^			200	200	200	200	200
Wheat meal ^2^			110	110	110	110	110
Corn starch ^2^			75	75	75	75	75
Fish oil ^3^			125	125	125	125	125
Vitamin–Mineral ^4^			20	20	20	20	20
Total			1000	1000	1000	1000	1000
Gross composition (g kg^−1^ DM)							
	AFM	ITTM	ITT_0_	ITT_25_	ITT_50_	ITT_75_	ITT_100_
Protein	67.02	66.4	384.0	388.7	389.4	382.4	381.8
Lipid	13.76	14.8	155.3	153.9	154.7	151.4	153.2
Ash	5.44	4.8	111.7	122.1	136.6	120.12	134.7
Arginine	3.16	3.48	1.62	1.65	1.60	1.57	1.70
Threonine	2.98	6.06	2.23	2.20	2.36	2.41	2.62
Tryptophan	2.02	3.12	1.98	2.02	2.33	2.40	2.57
Histidine	1.50	0.27	0.69	0.68	0.68	0.58	0.53
Valine	2.47	0.89	2.29	2.26	2.22	1.72	1.22
Methionine	1.05	1.01	0.95	0.93	0.98	0.90	0.90
Phenylalanine	1.42	2.40	1.88	1.99	1.99	2.02	2.04
Isoleucine	1.63	2.01	1.57	1.67	1.73	1.75	1.74
Leucine	0.31	2.87	1.84	1.91	1.97	1.95	1.99
Lysine	6.83	10.67	1.57	1.97	2.07	2.85	2.91

^1^ Anchovy fish meal. Koptur Balıkçılık. Trabzon.Turkey; ^2^ Soybean meal. Agromarin Yem San. ve Tic. A.Ş. İzmir.Turkey; ^3^ Anchovy fish oil. Agromarin Yem San. ve Tic. A.Ş. İzmir.Turkey; ^4^ Vitamin Mixture: Vitamin A. 18,000 IU kg^−1^ feed; Vitamin D3. 2500 IU kg^−1^ feed; Vitamin E. 250 mg kg^−1^ feed Vitamin K3. 12 mg kg^−1^ feed; Vitamin B1. 25 mg kg^−1^ feed; Vitamin B2. 50 mg kg^−1^ feed; Vitamin B3. 270 mg kg^−1^ feed; Vitamin B6. 20 mg kg^−1^ feed; Vitamin B12. 0.06 mg kg^−1^ feed; Vitamin C. 200 mg kg^−1^ feed; Folic acid. 10 mg kg^−1^ feed; Calcium d–pantothenate. 50 mg kg^−1^ feed; Biotin. 1 mg kg^−1^ feed; Inositol. 120 mg kg^−1^ feed; Choline chloride. 2000 mg kg^−1^ feed; Mineral Mixture (mg kg^−1^): Fe. 75.3 mg; Cu. 12.2 mg; Mn. 206 mg; Zn. 85 mg; I. 3 mg; Se. 0.350 mg; and Co. 1 mg.

**Table 2:** Primer sequences used in this study.

Gene	Oligonucleotide Sequence	Product Size (bp)	Gene Bank No.
β-actin	F: GCCGCGACCTCACAGACTACC	126	NP_001117707.1
	R: CAAAGTCCAGCGCCACGTAGCA		
IL1-β	F: GGAGAGGTTAAAGGGTGGCGA	106	AJ223954
	R: TGCCGACTCCAACTCCAACA		
IL-8	F: GAATGTCAGCCAGCCTTGTC	226	AJ279069.1
	R: TCCAGACAAATCTCCTGACCG		
TNF-α	F: GGGGACAAACTGTGGACTGA	1056	NM_001124357.1
	R: GAAGTTCTTGCCCTGCTCTG		
GH	F: GTACCCTAGCCAGACCCTGATC	5508	NM_001124689
	R: TCTTGAAGCAAGCCAACAACTC		
IGF-1	F: TGGACACGCTGCAGTTTGTGTGT	863	M95183
	R: CACTCGTCCACAATACCACGGT		

**Table 3:** Growth performance, sustainability and economic assessment of rainbow trout fed with experimental diets for 60 days.

	ITTM_0_	ITTM_25_	ITTM_50_	ITTM_75_	ITTM_100_
Initial weight (g)	64.62 ± 1.35	66.77 ± 1.60	66.13 ± 0.92	66.08 ± 0.07	65.47 ± 1.38
Final weight (g)	132.20 ± 2.80 ^b^	147.44 ± 1.17 ^a^	145.31 ± 1.30 ^a^	123.89 ± 1.84 ^c^	106.78 ± 2.99 ^d^
Relative growth rate (%)	104.60 ± 3.90 ^b^	120.86 ± 3.58 ^a^	119.75 ± 3.80 ^a^	87.46 ± 3.00 ^c^	63.09 ± 2.12 ^d^
Specific growth rate (% day^−1^)	1.19 ± 0.03 ^b^	1.32 ± 0.02 ^a^	1.31 ± 0.03 ^a^	1.04 ± 0.03 ^c^	0.81 ± 0.02 ^d^
Feed conversion	0.88 ± 0.03 ^c^	0.74 ± 0.01 ^d^	0.75 ± 0.02 ^d^	1.03 ± 0.03 ^b^	1.45 ± 0.06 ^a^
Feed cost (US $/kg diet)	1.36	1.32	1.27	1.22	1.17
FCE	1.13 ± 0.04 ^b^	1.34 ± 0.01 ^a^	1.32 ± 0.03 ^a^	0.96 ± 0.03 ^c^	0.69 ± 0.03 ^d^
FMU	417.61 ± 14.02 ^a^	262.20 ± 1.51 ^b^	178.10 ± 3.78 ^c^	122.06 ± 3.97 ^d^	0.00 ± 0.00 ^e^
FOU	111.07 ± 3.73 ^c^	91.97 ± 0.53 ^d^	94.75 ± 2.01 ^d^	129.85 ± 4.22 ^b^	181.80 ± 8.23 ^a^
ECR	1.20 ± 0.04 ^b^	0.98 ± 0.08 ^c^	0.92 ± 0.02 ^c^	1.27 ± 0.04 ^b^	1.70 ± 0.08 ^a^
EPI	0.18 ± 0.01 ^b^	0.22 ± 0.01 ^a^	0.22 ± 0.01 ^a^	0.16 ± 0.01 ^c^	0.12 ± 0.01 ^d^
PRO	2.79 ± 0.04 ^b^	3.02 ± 0.01 ^a^	3.08 ± 0.02 ^a^	2.73 ± 0.04 ^b^	2.30 ± 0.08 ^c^

*n* = 3X ± SD, means with different alphabetical characters in the same row are statistically different (*p* < 0.05). FCE—feed conversion efficiency (g/g), FMU—relative fish meal use (g/L kg of fish gain), FOU—relative fish oil use (g/L kg of fish gain), FC—feed cost ($/kg), ECR—economic conversion ratio ($/L kg of fish gain), EPI—economic profitability index ($/fish), and PRO—profitability ($/L kg of fish gain).

**Table 4:** Fillet proximate composition and amino acid profile of rainbow trout fed with experimental diets for 60 days.

	ITTM_0_	ITTM_25_	ITTM_50_	ITTM_75_	ITTM_100_
Moisture (%)	74.68 ± 0.16	74.51 ± 0.65	74.48 ± 0.61	75.21 ± 0.28	75.50 ± 0.47
Crude protein (%)	22.91 ± 0.65 ^a^	20.15 ± 0.97 ^b^	20.60 ± 0.54 ^b^	19.28 ± 0.78 ^b^	19.64 ± 0.66 ^b^
Crude lipid (%)	0.58 ± 0.20 ^b^	2.66 ± 0.30 ^a^	2.28 ± 0.44 ^a^	2.83 ± 1.06 ^a^	2.86 ± 0.73 ^a^
Crude ash (%)	1.60 ± 0.08	1.95 ± 0.26	1.66 ± 0.01	1.85 ± 0.35	1.62 ± 0.13
Amino acid concentration (% of protein)					
Arg (Arginine)	1.32 ± 0.02	1.18 ± 0.05	1.14 ± 0.01	1.30 ± 0.16	1.16 ± 0.01
His (Histidine)	0.64 ± 0.02	0.59 ± 0.07	0.57 ± 0.01	0.56 ± 0.07	0.60 ± 0.02
Ile (Isoleucine)	1.84 ± 0.05	1.80 ± 0.01	1.51 ± 0.06	1.68 ± 0.24	1.60 ± 0.21
Leu (Leucine)	1.02 ± 0.03 ^ab^	1.08 ± 0.01 ^a^	0.91 ± 0.02 ^b^	0.98 ± 0.11 ^ab^	0.95 ± 0.02 ^ab^
Lys (Lysine)	1.87 ± 0.02 ^a^	1.91 ± 0.01 ^a^	1.46 ± 0.02 ^b^	1.54 ± 0.26 ^ab^	1.57 ± 0.21 ^ab^
Met (Methionine)	0.71 ± 0.06	0.65 ± 0.05	0.56 ± 0.09	0.73 ± 0.08	0.61 ± 0.03
Phe (Phenylalanine)	0.75 ± 0.03	0.75 ± 0.02	0.65 ± 0.01	0.71 ± 0.08	0.68 ± 0.06
Thr (Threonine)	11.54 ± 0.25	10.65 ± 0.41	10.78 ± 0.31	10.56 ± 0.45	10.71 ± 0.83
Val (Valine)	2.95 ± 0.06 ^a^	2.87 ± 0.02 ^a^	2.41 ± 0.12 ^b^	2.62 ± 0.13 ^ab^	2.39 ± 0.20 ^b^
∑EAA	22.66 ± 0.39 ^a^	21.44 ± 0.36 ^ab^	20.01 ± 0.27 ^b^	20.69 ± 0.71 ^b^	20.28 ± 0.79 ^b^
Ala (Alanine)	15.89 ± 0.06	17.40 ± 0.26	17.52 ± 0.46	15.22 ± 0.36	15.23 ± 2.43
Asp (Aspargine + Aspartic acid)	17.46 ± 0.40	15.28 ± 0.23	15.59 ± 0.32	15.97 ± 0.79	15.89 ± 2.34
Cys (Cystine)	0.10 ± 0.00	0.10 ± 0.00	0.09 ± 0.00	0.11 ± 0.01	0.10 ± 0.00
Glu (Glutamic acid)	29.45 ± 0.76	31.16 ± 0.18	30.85 ± 1.24	30.74 ± 0.43	30.05 ± 1.33
Gln (Glutamine)	2.06 ± 0.05 ^a^	2.02 ± 0.02 ^ab^	1.71 ± 0.06 ^b^	1.82 ± 0.08 ^ab^	1.83 ± 0.23 ^ab^
Pro (Proline)	4.83 ± 0.05 ^a^	3.79 ± 0.15 ^b^	3.66 ± 0.21 ^b^	4.05 ± 0.19 ^b^	4.05 ± 0.23 ^b^
Ser (Serine)	7.62 ± 0.30 ^b^	7.97 ± 0.02 ^ab^	8.05 ± 0.19 ^ab^	8.82 ± 0.34 ^a^	6.52 ± 0.55 ^c^
Tyr (Tyrosine)	0.74 ± 0.04	0.65 ± 0.01	0.63 ± 0.01	0.67 ± 0.08	0.67 ± 0.06
∑NEAA	78.16 ± 1.45	78.0.03	78.12 ± 1.94	77.40 ± 1.47	74.35 ± 4.79
∑EAA/∑NEAA	0.29 ± 0.00 ^a^	0.27 ± 0.00 ^ab^	0.25 ± 0.00 ^b^	0.26 ± 0.00 ^ab^	0.27 ± 0.02 ^ab^

Means with different alphabetical characters in the same row are statistically different (*p* < 0.05).

**Table 5:** Hematological and serum biochemical parameters of rainbow trout, *O. mykiss,* fed diets containing different levels of ITTINSECT™ APS V1 meal (ITTM) for 60 days.

Groups	Rbc (×10^6^ per mm^3^)	Hct (%)	Hgb (g/dL))	TPROT (g/dL)	ALB (g/dL)	GLO (g/dL)	GLU (mg/dL)	CHOL (mg/dL)	TRIG (mg/dL)
ITTM_0_	1.46 ± 0.21	25.15 ± 3.20	8.80 ± 0.77	11.54 ± 1.26 ^a^	0.80 ± 0.06	10.74 ± 1.23 ^a^	81.08 ± 12.93 ^b^	95.10 ± 27.7	37.63 ± 12.28
ITTM_25_	1.37 ± 0.13	24.60 ± 3.45	8.03 ± 1.09	12.25 ± 0.98 ^a^	0.78 ± 0.13	12.30 ± 1.80 ^a^	82.20 ± 16.83 ^b^	105.47 ± 11.31	35.16 ± 7.26
ITTM_50_	1.46 ± 0.09	24.95 ± 2.15	7.98 ± 0.48	10.87 ± 1.98 ^ab^	0.85 ± 0.21	9.95 ± 1.88 ^ab^	91.84 ± 17.58 ^ab^	100.53 ± 7.54	41.15 ± 3.86
ITTM_75_	1.32 ± 0.12	22.12 ± 1.84	7.71 ± 0.56	8.77 ± 0.91 ^b^	0.73 ± 0.23	6.73 ± 2.31 ^b^	114.30 ± 15.62 ^a^	116.80 ± 7.28	37.86 ± 6.81
ITTM_100_	1.41 ± 0.19	23.60 ± 3.47	7.77 ± 0.79	10.80 ± 1.63 ^ab^	0.64 ± 0.17	10.16 ± 1.48 ^a^	101.47 ± 14.18 ^ab^	102.05 ± 19.63	43.67 ± 7.57

The values (*n* = 15) are mean ± standard deviation with common superscripts in the same line are significantly different (*p* < 0.05). RBC. red blood cell; Hct. hematocrit; Hb. hemoglobin; GLU. glucose; TPROT. total protein; COL. cholesterol; ALP. alkaline phosphatase; GOT. glutamic oxaloacetic transaminase; and GPT. glutamic pyruvic transaminase.

## Data Availability

The data presented in this study are available on request from the corresponding authors.
